# Supplementary material for: Patterns of Intron Gain and Loss in Fungi
Source: PLoS Biol. 2004 Nov 30;2(12):e422. doi: 10.1371/journal.pbio.0020422 (PMC532390; doi:10.1371/journal.pbio.0020422)
Supplement: Table S1 — Also available at http://genes.mit.edu/NielsenEtAl/. (4.3 MB ZIP). [file pbio.0020422.st001.zip › NielsenEtAl/html/1426.html]

AN3973.1.NCU06031.1.MG08256.1.FG07536.1


```
 CLUSTAL W (1.82) Multiple Sequence Alignments - Introns Inserted


Sequence 1: NCU06031.1	225 aa
Sequence 2: MG08256.1	213 aa
Sequence 3: FG07536.1	226 aa
Sequence 4: AN3973.1	261 aa
Alignment Length: 269 aa
Number Identitical Residues: 125 aa
Alignment Score (without introns) 6009


MG08256.1 	-------------------------------------------------~--MGTEA~PN
NCU06031.1	----------------------------------------MASTDRNAP2LRLGTIA~PN
FG07536.1 	----------------------------------------MSAP---AP~LRLGSTA~PN
AN3973.1  	MASFFSRTTFRTLNTLFKATPCSKTPITAPLAQPLARRFIGTLPREQPR~LRLGSTA1PN
          	 ::  : ::  : .:  .::..:.:. ::. :.. :     : .  ..    :*: * **

MG08256.1 	FKAETTKGPIDFHEFIGSNWVILFSHPEDFTPVCTTELGEFARLEPEFTKRGVKLIGL~S
NCU06031.1	FQADTTTGPIDFHEFIGDNWVILFSHPEDYTPVCTTELGEMARLEPEFKKRGVKLIGL~S
FG07536.1 	FQAETTKGNIDFHEFIGDNWVILFSHPEDYTPVCTTELGAFAKLQPEFTKRNVKLIGL~S
AN3973.1  	FEALTTQGKIDFHQWMDGCWTILFSHPADFTPVCTTELGAFAKLKGEFAKRGVKMIGL0S
          	*:* ** * ****:::.. *.****** *:********* :*:*: ** **.**:*** *

MG08256.1 	ANTVGSHDGWIKDINDVTGSHVAFPIIADKERKVAYLYDM~LDYQDTTNVDEKGIAFTIR
NCU06031.1	ANTLGSHEGWINDIKDVTGSQVQFPIIADKERKVAYLYDM2LDYQDTTNVDEKGIAFTIR
FG07536.1 	ANTIQSHEGWIKDIGEVTGGNVEFPIIGDKERKVSLLYDM2IDQQDATNVDEKGIAFTIR
AN3973.1  	ANDLGSHGDWIKDINEVTGSDVQFPIIADADRKVAWLYDM~IDESELANLAEKGIAFTIR
          	** : ** .**:** :***..* ****.* :***: **** :* .: :*: *********

MG08256.1 	SVFIIDPAKKIRTILSYPASTGRNSAEVLRIVDSLQTGDKHKVTTPINWVPGDDVIVHPS
NCU06031.1	SVFVIDPKKTIRTILAYPASTGRNSAEILRIVDSLQTGDKHKVTTPINWVPGDDVIVHPS
FG07536.1 	SVFIIDPKKTIRTIFSYPASTGRNAAEVLRVIDSLQTGDKYRITTPINWVPGEDVIVHPS
AN3973.1  	SVFIIDPAKKIRLVMSYPASTGRNTAEVLRVIDSLQTADKKGIATPIDWQVGDDVIVPPS
          	***:*** *.** :::********:**:**::*****.**  ::***:*  *:**** **

MG08256.1 	IKDEQAKDLFPNFRAVKPYLRFTPLPKE-----
NCU06031.1	IKGEEATRLFPNLKAVKPYLRFTPLPKDA----
FG07536.1 	VKNEEAKTLFPEFRIVKPYLRFTPLAKEKVLPQ
AN3973.1  	VSTEDAKKKFGEVRELKPYLRYTKY--------
          	:. *:*.  * :.: :*****:*
```
